# Clonal Reconstruction of Thyroid Cancer: An Essential Strategy for Preventing Resistance to Ultra-Precision Therapy

**DOI:** 10.3389/fendo.2019.00468

**Published:** 2019-07-18

**Authors:** Elizabeth R. McGonagle, Carmelo Nucera

**Affiliations:** ^1^Division of Experimental Biology, Laboratory of Human Thyroid Cancers Preclinical and Translational Research, Department of Pathology, Cancer Research Institute (CRI), Center for Vascular Biology Research (CVBR), Beth Israel Deaconess Medical Center, Harvard Medical School, Boston, MA, United States; ^2^Broad Institute of Harvard and MIT, Cambridge, MA, United States

**Keywords:** ultra-precision therapy, BRAF^V600E^, microenvironment, vemurafenib, CDK4/6, palbociclib, copy number variations, clonal evolution

## Abstract

The introduction of ultra-precision targeted therapy has become a significant advancement in cancer therapeutics by creating treatments with less off target effects. Specifically with papillary thyroid carcinoma (PTC), the cancer's hallmark genetic mutation BRAF^V600E^ can be targeted with selective inhibitors, such as vemurafenib. Despite initial positive tumor responses of regression and decreased viability, both single agent or combination agent drug treatments provide a selective pressure for drug resistant evolving clones within the overall heterogeneous tumor. Also, there are evidences suggesting that sequential monotherapy is ineffective and selects for resistant and ultimately lethal tumor clones. Reconstructing both clonal and subclonal thyroid tumor heterogeneous cell clusters for somatic mutations and epigenetic profile, copy number variation, cytogenetic alterations, and non-coding RNA expression becomes increasingly critical as different clonal enrichments implicate how the tumor may respond to drug treatment and dictate its invasive, metastatic, and progressive abilities, and predict prognosis. Therefore, development of novel preclinical and clinical empirical models supported by mathematical assessment will be the tools required for estimating the parameters of clonal and subclonal evolution, and unraveling the dormant vs. non-dormant state of thyroid cancer. In sum, novel experimental models performing the reconstruction both pre- and post-drug treatment of the thyroid tumor will enhance our understanding of clonal and sub-clonal reconstruction and tumor evolution exposed to treatments during ultra-precision targeted therapies. This approach will improve drug development strategies in thyroid oncology and identification of disease-specific biomarkers.

## Next Step to Ultra-Precision Targeted Therapies in Thyroid Carcinoma

Papillary thyroid carcinoma (PTC) is the most frequent type of thyroid cancer, accounting for almost 80% of thyroid cancer cases. The genetic hallmark of human PTC is the somatic BRAF^V600E^ mutation. Constitutive activation of the BRAF proto-oncogene (e.g., valine to glutamic acid substitution, V600E) leads to activation of ERK1/2 pathway and other intracellular pathways associated with abnormal cell proliferation and growth (1–3).

BRAF^V600E^ can be targeted with selective inhibitors such as vemurafenib, the first FDA approved orally administered inhibitor (4). Inhibition of the BRAF^V600E^ oncogene has shown promising results for the suppression of the BRAF-ERK1/2 pathways and an overall regression in tumor growth (5). Concurrently, vemurafenib has also been shown to aid in cancer cell redifferentiation for increased iodide incorporation in RAIR, BRAF^V600E^ patients (6). However, despite early clinical success with vemurafenib treatment, this drug therapy can generate positive selection for drug resistant cancer clones (7), and disease progression is observed in the clinic (8–10). Studies consistently show that BRAF^V600E^-cancer cells go into cell cycle arrest upon vemu treatment, but with continued exposure exhibit a rebound of pERK1/2 and resume proliferation (7, 11–14).

Given cancer's hallmark of “enabling replicative immortality,” unregulated replication creates greater possibility for mutations to arise and succeed in the cell population, since most cancer cells dedifferentiate and require activation of angiogenic, invasive, and metastatic genes (15). Each tumor is comprised of a diverse number of cells, each with a unique genetic profile. The unique genetic profile of each cancer cell means within any given tumor there is a diverse range of tumor clones with diverse cell clusters each containing different mutations.

Cancer is considered a “disease of the genome,” whereby a variety of genetic mutations leads to individual cells that proliferate and divide without regard to their internal regulatory mechanisms (16). Cancer's deregulation of proliferation allows for a greater possibility for genetic mutations to occur during mitotic division, as well as the greater chance that gene mutations will not trigger cellular death pathways. Mutations that do not induce apoptosis be they somatic copy number mutations, insertions, deletions, or whole chromosome amplifications, create a heterogeneous population of tumor cells. Over an entire tumor, each individual cell is undergoing its own dysfunctional replication and acquiring its own unique mutations. It is throughout a tumor's progression that some populations of cells with mutations, that increase the tumor's fitness to its environment, are selected. Indeed, an important characteristic of cancer is its “evolutionary character,” which in many ways should be considered an additional hallmark of the disease (17). Tumor cells are composed of an uniform clonal composition for driver mutations. Driver mutations, mostly acquired somatically, confer a growth advantage to the tumor, enabling outgrowth of neoplastic clones and contributing to tumor progression (18).

Throughout a tumor cell's evolution, the cell has to evolve many different mechanisms to prevent induction of cell death (e.g., apoptosis) and suppression of growth signals. Part of a cell's evolution to evade these signals involves genetic mutations, which predisposes the cells to being able to survive with mutations that would otherwise not survive in non-cancerous or malignant cells. Tumorigenesis requires increased mutability in order for tumor cells to bypass regulatory and apoptotic pathways and signals. Increased mutability of cells is achieved through down regulation of genomic maintenance mechanisms, including P53 gene inactivation (19). The down regulation of genomic maintenance creates a foundation for cancer cells to acquire new genetic mutations and be susceptible to mutagens.

Cancer's evolution is not solely dictated by individual tumor cell's irregular proliferation conferring gains in somatic mutations. A tumor's microenvironment, along with individual cancer cell genome instability, encourages clonal expansion and heterogeneity (20). While each cancer cell could contain a hallmark driver mutation, such as BRAF^V600E^ in PTC (21), these PTC cells are likewise able to diversify through different subclonal genetic alterations. The driver or classical genetic biomarker would be a mutation at the clonal level which leads to the progression of those cells to become cancerous. Nonetheless, these cells are still dividing and proliferating, albeit irregularly, and are able to gain somatic mutations. So in addition to their driver mutation, tumor cells are evolving multiple subclonal mutations that can be positively selected for just as their primary driver mutation was initially and might impact prognosis and clinical outcome (22).

The microenvironment is able to exert selective pressures on the tumor, allowing for positive selection of those clones with underlying subclonal mutations which are better suited to the changing microenvironment. Normal cells exhibit a behavior referred to as “fitness sensing” whereby cells compete with neighboring cells, and those cells that are less fit compared to their neighbors die (23). In the case of a tumor with subclonal heterogeneity, intercellular competition between cells could aid in the positive selection of resistant clones driven by interaction in the tumor's microenvironment. Genetically diverse cells may not be competing with each other but also interacting with each other (24, 25). Consequently tumors could be increasing their genetic complexity and heterogeneity by allowing multiple tumor subclones to survive within the overall tumor environment and to interact and influence one another.

Within a recurring tumor repopulated maybe by cells containing vemurafenib (FDA-approved, selective inhibitor of BRAF^V600E^) resistant subclones (8), intercellular competition could occur between different subclones, making those selected cells “super competitor cells.” This type of subclonal intercellular competition could potentially select subclones that confer the greatest possible resistance, invasive, and metastatic abilities to the tumor.

While cancer cells undergo a form of gradualism in terms of their clonal expansion, targeted drug therapy can be understood as creating a punctuated equilibrium event within the life of a tumor (26). For example, in patients with BRAF^V600E^ positive melanoma upon treatment with vemurafenib therapy (an example of ultra-precision therapy), some tumor cells will respond to the treatment and die (27). However, those cells containing advantageous subclonal mutations, in addition to BRAF^V600E^, will be able to overcome inhibition of BRAF^V600E^ and subsequently of the ERK1/2 intracellular signaling, and will continue to survive and proliferate. Subclones that are able to survive drug therapy are aided by a process called “competitive release,” where not only do passenger mutations allow individual cells to survive treatment, but any potential competing cells have been killed through drug therapy (28). In this way, tumor undergoes a punctuated equilibrium event, facilitated by targeted drug therapy, where those cells with selectively advantageous subclonal mutations rapidly become the majority of the cells composing the tumor.

While all of the cells of a single tumor may contain a genetic biomarker mutation, such as BRAF^V600E^ in PTC, the individual tumor clones may contain additional genetic alterations as subclonal genetic event. Treatment of a tumor with vemurafenib will suppress the BRAF^V600E^ mutation, eliciting cytostatic effects on tumor growth inhibition (7, 12, 29), but cell populations within any tumor that have subclonal genetic alterations could bypass the inhibition of oncogenic pathways by targeted therapy and still allow the tumor cell to grow (30). These tumor subclones, under the exacerbated selective pressure of targeted therapy will evoke in the tumor drug resistance, will survive and could eventually lead to tumor progression (31).

**Figure 1:**
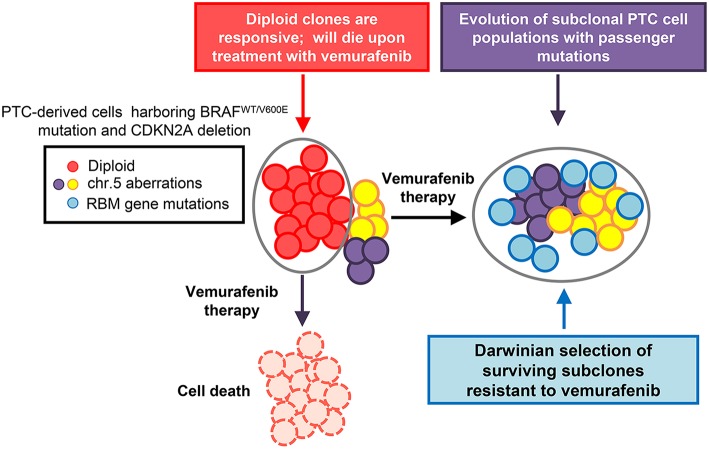
Model of thyroid carcinoma clonal evolution. PTC-derived KTC1 cells clonally harbor the hallmark heterozygous BRAF^WT/V600E^ mutation and show loss of the CDKN2A (P16) gene. The diploid clones compose the majority of the tumor cells genetic landscape; with there being other subclonal populations present within the overall tumor. Upon treatment with targeted therapy, in this case using the selective BRAF^V600E^ inhibitor, vemurafenib, diploid KTC1 clones will respond positively to the drug treatment and die. However, those clones containing additional subclonal mutations, such as mutations in RBMX and RBM10 genes, or chromosome 5p amplifications, are able to overcome drug induced suppression of the BRAF^V600E^ pathway. Through the course of vemurafenib treatment, subclones with passenger mutations, such as RBM gene family somatic mutations, can be positively selected. This tumor geography will then become enriched with populations of drug resistant mutant clones. This tumor composed primarily of vemurafenib-resistant clones, might be effectively untreatable and has the potential to lead to tumor progression.

Specifically in PTC-derived cells harboring the heterozygous BRAF^V600E^ mutation and with loss of the CDKN2A (P16) gene, targeted drug treatment therapy positively selected for a number of drug resistant clones and drove tumor clonal evolution (Figure 1) (7). In this study, PTC-derived cells harboring the BRAF^V600E^ mutation were treated with both vemurafenib and palbociclib. Palbociclib is an FDA approved CDK4/6 inhibitor typically used to treat patients with advanced stage HER2 negative breast cancer (32). Single treatment therapy with vemurafenib on BRAF^V600E^ and P16^−/−^ thyroid tumor cells lead to a clonal expansion of treatment resistant clones, showing chromosome 5 amplifications and RBM (RNA-binding motif) gene family mutations (7). Importantly, combined therapy with vemurafenib and palbociclib was able to ameliorate either primary or secondary drug resistance in these tumor cells by inducing cell death and inhibiting the adaptive subclonal evolution (7).

Combination therapy is seen as the best treatment option for overcoming resistance to single agent targeted therapy (Table 1). However, combination therapy also has the potential to drive tumor recurrence by selecting subclones that are resistant to both agents used in the dual therapy treatment (40). Statistical models highlight that tumor cells are much more likely to become resistant to dual therapy through the occurrence of one gene mutation conferring resistance to both drugs simultaneously than through sequential mutations conferring resistance to each drug separately (41).

**Table 1:** Targeted therapies in thyroid carcinoma.

**Therapeutic**	**Target(s)** **(Inhibitory effects)**	**Median progression free survival**
Vemurafenib	BRAF^V600E^	50% of patients with progression-free survival of ~18 months (33)
Dabrafenib	BRAF^V600E^	50% of patients with progression-free survival of ~11.3 months (34)
Sorafenib	Multi-kinase (including BRAF^WT^ and BRAF^V600E^)	~17.9 months (35)
Lenvatinib	VEGFR1-3, FGFR1-4, PDGFRα, PDGFRβ, KIT, RET (36–38)	~18.3 months (39)

Not only does targeted drug therapy have the potential to positively select cancer cells with resistant subclonal mutations, but could also enrich the population of cells carrying advantageous and potentially cancerous passenger mutations. One study showed that somatic mutations in the thyroglobulin (Tg) gene were associated with poorer clinical prognosis (42). Additionally, the study found that PTC samples with Tg mutations showed more distant metastasis compared with PTC samples that did not contain somatic Tg mutations. Given that most of the Tg mutated PTC samples also contained MAPK pathway mutations, such as BRAF^V600E^, Tg mutations were more likely passenger mutations that are advantageous in the malignant evolution of PTC as opposed to the actual drivers of PTC. Since Tg mutations are associated with mutations in genes encoding components of the MAPK signaling pathway, it is possible that some subclones harboring both driver and maybe passenger mutations, such as RBM gene family mutations (e.g., RBM10, RBMX) (7), could survive to vemurafenib treatment and allow for a recurring tumor that is more invasive and drug resistant (7).

Another experimental model focusing on tumor heterogeneity in breast cancers identified a subclonal genetic marker associated with CD44+ breast cancer stem cells. Previous studies had demonstrated that CD44+ cells displayed increased tumorigenesis compared to CD44– cells (43). Given this knowledge, researches began genetically profiling CD44+ breast cancer tumor cells and identified a CD44+ cell-specific gene, Protein C receptor (PROCR) (44). PROCR is a transmembrane protein commonly expressed in endothelial cells, and has been shown to have anticoagulation as well as anti-inflammatory functions (45). Importantly for this breast cancer model, PROCR has been previously noted to also be a marker for mammary epithelial stem cells (46). While not necessarily a passenger mutation, the discovery of the PROCR gene being specific to CD44+ breast cancer cells creates a better understanding of the cellular mechanisms and pathways activated in breast cancer. Understanding the relationship between the CD44 and PROCR genes allows for a better clonal reconstruction of the overall heterogeneous tumor and for the identification of ultra-precision therapy.

In the PTC experimental model, an understanding of the subclonal construction of the tumor having chromosome 5 amplifications and RBM gene family mutations, revealed potential pathways for secondary resistance. This knowledge of both the clonal and subclonal configuration of the tumor, allowed for a more effective solution to treating BRAF^V600E^ and CDKN2A^−/−^ mutant PTC's and for overcoming both innate/primary and secondary tumor resistance mechanisms.

**Figure 2:**
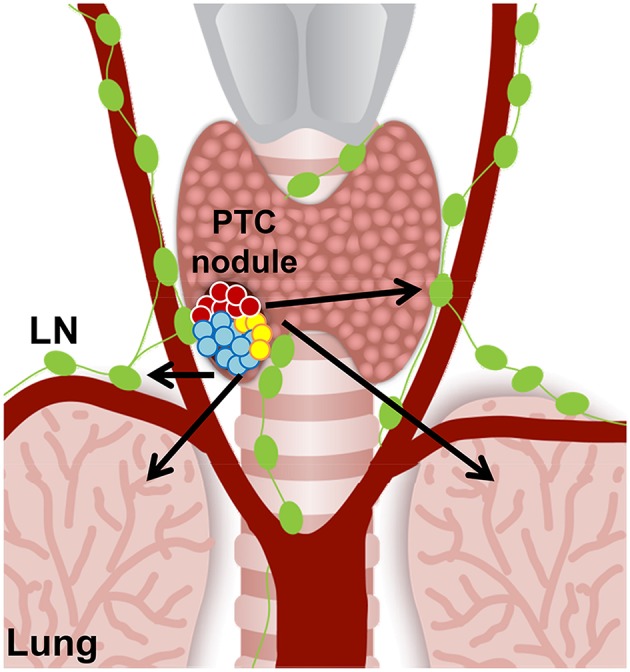
Hypothetical cell composition of a BRAF^WT/V600E^ papillary thyroid carcinoma (PTC) nodule. BRAF^WT/V600E^ positive malignant thyroid nodule with heterogeneous tumor cell clusters that might have the potential for metastasis (arrows) to the neck lymph nodes (LN) and lungs/pleura. Red circles: diploid PTC cells; aqua and yellow circles: PTC cells with aneuploidy.

Beyond elucidating better understandings of potential cellular pathways and genes involved in drug therapy resistance, clonal and subclonal reconstructions of tumors can also reveal biomarkers for early detection of aggressive thyroid cancer that could dictate future tumor behavior. With regard to thyroid cancers, PTC is classified as a differentiated thyroid cancer, and other types of thyroid carcinomas, such as anaplastic thyroid carcinoma (ATC), are classified as undifferentiated thyroid cancer. The transition from PTC to undifferentiated thyroid carcinoma is still poorly understood and furthermore effective treatments of ATC represent a large unmet clinical need. Clonal and subclonal reconstructions of both heterogeneous PTC (Figure 2) and ATC will strongly help to better understand the genes and cellular players involved in this malignant transition, and in the metastatic process. Given ATC's poor prognosis and limited treatment options, increased knowledge of subclonal reconstruction might allow for the development of better treatments and even potential targeted drug therapies.

Importantly, ATC show increased mutational burden compared to PTC. In one study examining the genetic profile of 196 ATC samples, only 41% (81 tumors) harbored BRAF mutations (47). If ATC arise from the high risk aggressive PTC, then they increase tumor mutational burden (47), suggesting that additional mutations may trigger tumor resistance and overcome any potential single agent ultra-precision targeted therapy. Intriguingly, increased tumor mutational burden has been correlated with a positive outcome using immunotherapy (48). Subclonal reconstruction could also help to solidify understanding of which biomarkers and mutations indicate responsiveness to immunotherapy as well as targeted therapy. An additional area of unmet clinical need is in understanding and treating metastatic thyroid tumors; distant metastases may occur early and remain dormant in the metastatic niche (49). Tumor dormancy refers to the phenomenon where a small cluster of cells, derived from the primary tumor itself, metastasize and are able to survive in a quiescent state for an extended period of time without growth (50). Since some tumor cells are in a quiescent state, these cells can survive without growing or developing a tumor for years; until the dormant cells are then activated by some mechanism, in which case invasive tumors may arise from these dormant cells. The importance in considering dormant metastatic tumors is in considering their potential subclonality. If these cells are early evolved subclones, they could potentially harbor secondary mutations, which either facilitated their quiescent state or that eventually allows them to overcome single agent targeted therapy. Progression of metastatic tumors aligns with the parallel progression theory of thyroid tumorigenesis, and the metastatic thyroid tumors may arise from the dormant cell populations. Additionally, thyroid carcinoma metastases exhibit tissue specificity; with PTC primarily metastasizing to the neck lymph nodes (and lesser in the lungs) and medullary thyroid carcinomas (MTC) migrating to the liver and bone (49). A clonal reconstruction of the dormant thyroid tumor cells may also broaden the understanding of why certain cells become metastatic and why certain metastases can be tissue specific. Dormancy is thought to be prompted by a lack (or impairment) of angiogenesis, so comprehensive clonal examination could likewise illuminate genes and markers of the angiogenic balance (51).

In sum, some bioinformatics models have been designed and are being used to reconstruct the clonal and subclonal heterogeneity of tumors. However, we need novel experimental models performing the clonal and subclonal reconstruction both pre- and post-drug treatment(s) of the thyroid tumor for somatic mutational and epigenetic profile, copy number variation, cytogenetic alterations, and non-coding RNA expression. Clonal and subclonal evolution of tumor cell clusters is also driven by the selective pressure of targeted drug therapy, therefore the pre-treatment reconstruction and the post-treatment reconstruction would identify all clones and subclones that drug treatments positively select, those that are responsive to drug treatments and die, and clones containing passenger genetic alterations conferring secondary tumor resistance.

## Author Contributions

CN: design. CN and EM: editing.

### Conflict of Interest Statement

The authors declare that the research was conducted in the absence of any commercial or financial relationships that could be construed as a potential conflict of interest.
